# Thermometry of photosensitive and optically induced electrokinetics chips

**DOI:** 10.1038/s41378-018-0029-y

**Published:** 2018-08-27

**Authors:** Feifei Wang, Lianqing Liu, Gongxin Li, Pan Li, Yangdong Wen, Guanglie Zhang, Yuechao Wang, Gwo-Bin Lee, Wen Jung Li

**Affiliations:** 1 State Key Laboratory of Robotics, Shenyang Institute of Automation, Chinese Academy of Sciences, 110016 Shenyang, China; 2 University of Chinese Academy of Sciences, 100049 Beijing, China; 3 Shenzhen Academy of Robotics, 518057 Shenzhen, China; 4 Key Laboratory of Advanced Process Control for Light Industry of the Ministry of Education, Institute of Automation, Jiangnan University, 214122 Wuxi, China; 5 Department of Power Mechanical Engineering, National Tsing Hua University, 30013 Hsinchu, Taiwan; 6 Department of Mechanical and Biomedical Engineering, , City University of Hong Kong, Kowloon Tong, Hong Kong China

## Abstract

Optically induced electrokinetics (OEK)-based technologies, which integrate the high-resolution dynamic addressability of optical tweezers and the high-throughput capability of electrokinetic forces, have been widely used to manipulate, assemble, and separate biological and non-biological entities in parallel on scales ranging from micrometers to nanometers. However, simultaneously introducing optical and electrical energy into an OEK chip may induce a problematic temperature increase, which poses the potential risk of exceeding physiological conditions and thus inducing variations in cell behavior or activity or even irreversible cell damage during bio-manipulation. Here, we systematically measure the temperature distribution and changes in an OEK chip arising from the projected images and applied alternating current (AC) voltage using an infrared camera. We have found that the average temperature of a projected area is influenced by the light color, total illumination area, ratio of lighted regions to the total controlled areas, and amplitude of the AC voltage. As an example, optically induced thermocapillary flow is triggered by the light image-induced temperature gradient on a photosensitive substrate to realize fluidic hydrogel patterning. Our studies show that the projected light pattern needs to be properly designed to satisfy specific application requirements, especially for applications related to cell manipulation and assembly.

## Optically Induced Electrokinetics Chips: Too hot for cells?

Researchers have undertaken systematic measurement of the temperature distribution and changes over time in an optically induced electrokinetic (OEK) chip, paving the way for wider application of OEK technologies. In recent years, the use of OEK-based technologies has rapidly expanded thanks to their superior qualities in allowing the manipulation and assembly of biological and non-biological entities from the micro- to the nanoscale. However, simultaneously introducing optical and electrical energy into an OEK chip may cause problematic temperature increases. A team headed by Wen Jung Li and Lianqing Liu at the Chinese Academy of Sciences, Shenyang, measured the temperature distribution in an OEK chip and found that temperature was affected by light color, total illumination area, proportion of lit areas and voltage amplitude–findings that will help expand the application of OEK technologies in different fields.

## Introduction

In the past two decades, optically induced electrokinetics (OEK)-based technologies have rapidly extended into research fields such as manipulation, fabrication and assembly on the micro/nanoscale because of their superior ability to provide flexible, dynamic, non-invasive, high-resolution, and high-throughput approaches compared to traditional modalities (e.g., electrophoresis, dielectrophoresis, optical tweezers, magnetic tweezers, and acoustic traps)^1^. In 1995, Mizuno et al. used a focused laser beam to trigger a microvortex in liquid films to which a high-frequency electric field was applied enabling DNA translation and stretching^2,3^. By adjusting the local conductivity of an indium tin oxide (ITO) electrode using ultraviolet (UV) illumination to control electrophoretic deposition, Hayward et al. achieved the assembly of colloidal particles with diameters from submicrometers to 2 μm in the lighted regions^4^. By replacing the focused laser beam with a programmable illumination system and applying a highly efficient photoconductive material, Chiou et al. demonstrated an optical image-driven dielectrophoresis technique, usually referred to as optoelectronic tweezers (OETs) or optically induced dielectrophoresis (ODEP), for the parallel high-resolution manipulation of cells or microparticles^5^. ODEP retains the dynamic addressability of optical tweezers and high-throughput material selectivity of traditional dielectrophoresis while requiring five orders of magnitude less optical intensity than optical tweezers^6^. In addition to ODEP, a series of OEK-based phenomena have been explored, such as optoelectrowetting^7^, light-actuated alternating current (AC) electroosmosis^8^, light-actuated AC electrothermal flow^9^, optically induced electrohydrodynamic instability^10,11^, and optically induced electrochemistry^12,13^. These methods have been widely used to manipulate single cells or DNA molecules^14^ in parallel^5^, concentrate and transport multiple *Escherichia coli* cells^15^, trap single-wall and multi-wall carbon nanotubes^16,17^, manipulate and separate semiconducting and metallic nanowires^18^, control local pH or chemical concentration^19,20^, and fabricate microlens arrays^21^ and semiconducting/metallic devices^12,13^.

The realization of these methods depends on ingenious applications of photosensitive electrodes that usually contain a photoconductive layer (e.g., hydrogenated amorphous silicon (a-Si:H)^5^, single-crystalline bipolar junction transistors^22^, or bulk-heterojunction polymers^23^) and a transparent ITO electrode. When integrated with another ITO counter electrode, this photosensitive electrode is usually referred to as an OEK chip^21,24^. Dynamic or programmable manipulation is commonly triggered through the optical images generated by a digital micromirror device (DMD)^5,8,20^, a projector^9,21,24^, or a liquid-crystal-based spatial light modulator^25,26^. The constructed light images are usually focused by objectives with different magnifications for different applications by considering, e.g., the operational field of view, resolution, and required minimum virtual electrodes^5,13,21^. The absorbance of incident photons in the photoconductive layer induces the generation of electron-hole pairs and phonons, causing local conductivity and temperature increases, respectively^27^. Through the exploration of these temperature gradients generated in OEK chips, optically controlled bubble microrobots have been shown to manipulate microbeads and assemble microblocks and cell-encapsulating hydrogel beads based on the optically induced thermocapillary effect^28,29^. Additionally, the Joule heating driven by the applied electric field increases the temperature in the OEK chip further, similar to the conditions of dielectrophoresis (DEP)-based manipulations^27,30^. For biological applications, small temperature differences may induce significant variations in cell behavior and activity and protein reactivity^30–32^. Irreversible cell damage or death can arise from either a short, sharp increase or a sustained moderate increase in temperature^33,34^. The buffer temperature needs to be controlled such that it does not exceed physiological conditions by more than 3 °C for prolonged amounts of time to avoid the generation of any kind of hyperthermic stress in the cells^30^. Though OEK-based micro/nano manipulation and fabrication have been widely studied, systematic spatial and temporal temperature increases on OEK chips induced by illumination color and pattern have not been reported. Studying the OEK chip thermal effect is essential to facilitate further temperature control for various applications.

Here, we present systematic measurements of the temperature distribution and changes over time in an OEK chip and a photoconductive substrate. The influences of light color, projected image shape, image size, light distribution, and applied AC parameters on the temperature increase have been explored using an infrared (IR) camera that enables contactless transient temperature profile measurement without interfering with the thermal performance of the original system. To reveal the temperature distribution inside the OEK chip, we perform simulations based on the experimental parameters with results that are highly consistent with the experimental observations. The temperature distribution in cells dispersed in different areas of the OEK chip is studied by simulation. As a representative application of the light image-induced temperature gradient on a photosensitive substrate, fluidic hydrogel patterning driven by optically induced thermocapillary flow is proposed.

## Materials and methods

### Experimental setup

**Fig. 1:**
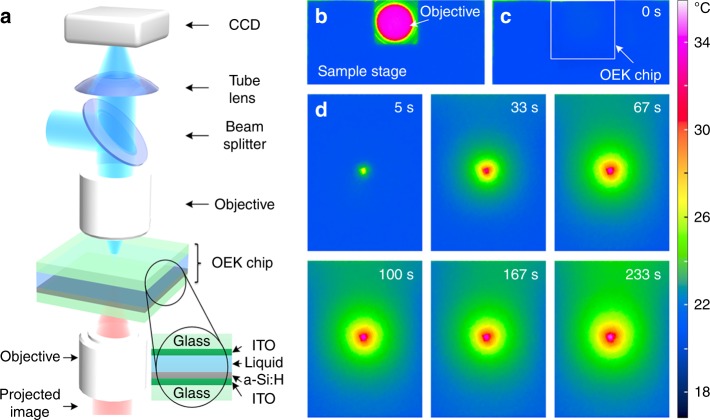
Temperature measurement of the OEK or PS chip. **a** Key experimental components used in the thermal imaging experiments. Temperature distribution on **b** the lower objective and **c**, **d** the OEK chip measured by an infrared camera. AC voltage with an amplitude of 20 *V*_pp_ and a frequency of 50 kHz was applied to the OEK chip. The OEK chip was illuminated by cyan-color light

The OEK chip photoconductive electrode used in our experiments is comprised of a 120 nm ITO layer sputtered onto 600-μm-glass and a ~1 μm a-Si:H layer deposited onto the ITO layer through plasma-enhanced chemical vapor deposition (PECVD). The thicknesses of the ITO and glass layers of the upper transparent ITO electrode are ~ 120 nm and ~ 1 mm, respectively. These two electrodes are separated by ~ 100-μm-thick double adhesive tape. The OEK chip is placed between a 50× objective (Nikon TU Plan EPI ELWD, Japan) focusing the light images generated by a commercial projector (VPL-F400X, Sony, Japan) and an optical microscope (Zoom 160, Optem, USA), as shown in Fig. 1a (schematic diagram) and Supplementary Figure S1 (actual photograph). Different colors generated by the projector are created by mixing different ratios of filtered red, green and blue light, which means the colors (e.g., “cyan” and “yellow”) are real. The photoconductive layer is placed at the working plane of the lower and upper objectives (Fig. 1a). The position of the OEK chip is adjusted by a three-dimensional translation platform (Leetro Automation Co. Ltd, China). When the chip temperature distribution is being monitored, the optical microscope is replaced by an IR camera (ImageIR4320, InfraTec GmbH, Germany), as shown in Supplementary Figure S1. The AC bias potential applied on the two ITO layers of the OEK chip is supplied by a function generator (Agilent 33522A, USA). For clarity, the OEK chip is called a photosensitive (PS) chip when the amplitude of AC potential is set to zero, i.e., a special case of OEK. Before the experiments, we first compare the hotplate temperature measured by the IR camera and a thermocouple sensor (#6212158, #5120288, RS Components Ltd) at several set points. The results show that these two methods are highly consistent (Supplementary Figure S2).

## Results and discussion

### Objective temperature

**Fig. 2:**
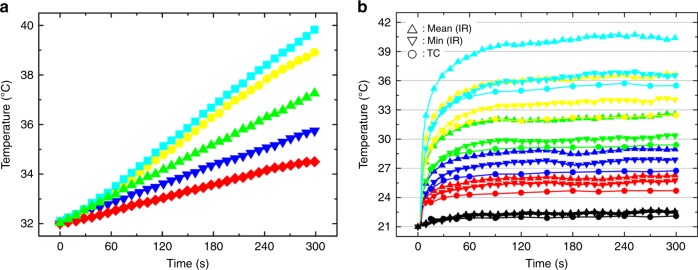
**a** Temperature of the objective illuminated by light with different colors. **b** Temperature changes of photoconductive substrate with the illumination time and colors monitored by an infrared (IR) camera or a thermocouple (TC) sensor. Different line colors represent different illumination colors

In our experiments, the objective working distance used to focus the projected images is ~10 mm, which may be close enough to heat the chip. The situation is worsened as the objective magnification increases or the working distance decreases. Therefore, we first examine the objective temperature increase upon illumination with projected light of different colors, such as red, blue, green, yellow and cyan with the spectrum shown in Supplementary Figure S3a. The projected images fill the rear objective aperture with single color light. The temperature distribution around the objective lens is shown in Fig. 1b. The average temperature in the middle area of the front objective lens is used to represent the objective temperature (the red region in Fig. 1b). Initially, the light color is adjusted to black, corresponding to the standby situation before manipulation, and the objective temperature gradually increases to a steady temperature of ~32 °C after illumination for ~1 h (Supplementary Figure S4a). The objective temperature increases as the light color is adjusted from black to other colors, and the rate of increase changes with the illumination color (Fig. 2a). When the projector is turned on and off with a switch duration of 150 or 60 s, the objective temperature shows evident thermal inertia (Supplementary Figure S4b, c).

### Photoconductive substrate temperature

Before studying the OEK chip, we first measured the temperature distribution and changes in the a-Si:H photoconductive substrate. When the objective reaches a steady temperature (~32 °C) under black illumination, the photoconductive substrate is directly placed on the pre-adjusted three-dimensional translation platform, enabling it to be laid on the focal plane of the lower objective (Fig. 1c). When the rear objective aperture is filled with light in our experimental setup, the focused light image on the photoconductive substrate is a solid circle with a diameter of ~773 μm. The size of this area is denoted S_full_. The average temperature extracted from this area is used for further analysis. As the photoconductive substrate is illuminated with light, the temperature increases quickly in the focus area (Fig. 2b) with a faster response than the objective (Fig. 2b and Supplementary Figure S4b). A net temperature increase of up to 20 °C from an initial temperature of ~21 °C is measured when the photoconductive substrate is illuminated with cyan light. The temperature measured by the thermocouple sensor is smaller than the minimum temperature detected by the IR camera (Fig. 2b), which may arise from the disruption of the thermal condition by introducing the thermocouple sensor. The diameter of the thermocouple sensor sensing unit is ~600 μm, which matches the illumination area. The photoconductive substrate optically induced thermal response is faster than the objective (Supplementary Figure S4 and S5), indicating that the temperature change in the substrate is mainly induced by the absorption of the projected light and that this illumination setup represents an efficient means of adjusting the temperature or establishing a temperature gradient.

When the photoconductive substrate is illuminated by projected light patterns with the same size but different shapes, the average temperature does not show an apparent reliance on the light pattern shapes (Supplementary Figure S6). However, the average temperature does decrease as the light patterns shrink (Supplementary Figures S6 and S7). When the total light size is the same, the light distribution in a certain area does not have a clear influence on the measured temperature; however, the steady temperature decreases as the total illumination area or the dark area increases (Supplementary Figure S8). In other words, the average temperature increases with the ratio of lighted to total controlled areas.

### PS and OEK chip temperature

**Fig. 3:**
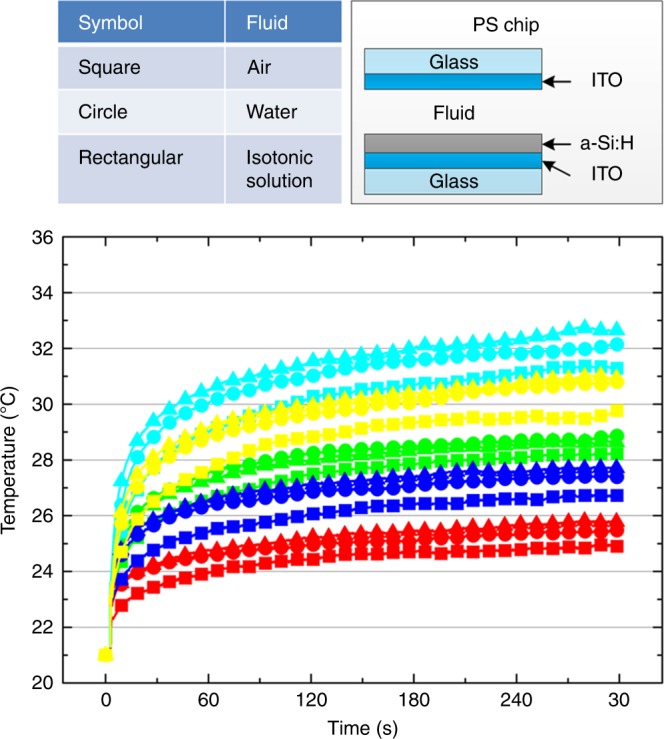
The relationship between temperature change in the PS chip and illumination time and color as monitored by an IR camera. Different color symbols represent different illumination colors. Squares, circles and rectangles, respectively, represent air, water and isotonic solution filled in the PS chip

**Fig. 4:**
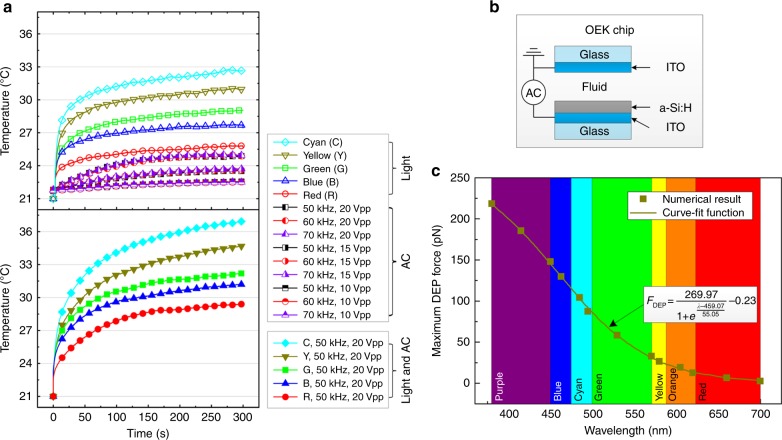
**a** Influence of illumination color and AC voltage on the temperature of OEK chips filled with an isotonic solution consisting of 8.5% (w/v) sucrose and 0.3% (w/v) glucose with conductivity of 1.3 × 10^−2^ S/m. **b** Schematic showing the OEK chip in these experiments. **c** The influence of illumination color on the DEP force applied to a 10 μm polystyrene bead

When the photoconductive electrode is encapsulated in PS chips filled with different fluids (air, deionized water, or isotonic solution), the measured temperature is less than the bare photoconductive electrode for each light color at the same detection time without applying AC voltage (Figs. 2, 3). Conversely, the OEK chip temperature increases with the applied AC amplitude but does not show an apparent dependence on the frequency when there is no illumination, which is consistent with prior analyses related to Joule heating in DEP manipulations^35^ (Fig. 4a). Without illumination, the maximum temperature increase induced by Joule heating in our experiments is ~2 °C when a 20 *V*_pp_ AC voltage is applied to the OEK chip, which is smaller than the temperature increase induced by light illumination (5–11 °C for different colored light). When illumination and AC voltage are applied to the OEK chip simultaneously, the temperature increases quickly and far exceeds the cases when they are used separately (Fig. 4a). Under these conditions, the light pattern increases the local conductivity of the photoconductive layer by triggering electron-hole pair generation, which transfers the AC voltage to the liquid layer and thus induces more Joule heating there ($q \propto \sigma \times U_{{\mathrm{rms}}}^2$, where *q* is the heating power per volume element, *σ* is the liquid conductivity, and *U*_rms_ is the root-mean-square voltage^35^).

Figure 4c shows that the maximum DEP force calculated by finite element method software (COMSOL) decreases with the optical wavelength increase according to the Fermi-Dirac function when the optical power is the same, which indicates that the ODEP force arises from electron-hole carrier generation in the photoconductive substrate^36^. Since both the temperature increase and DEP force rely on the wavelength, the optimized DEP force may not be best for biomanipulation, i.e., the best color for maximizing the DEP force may induce a serious temperature increase (Fig. 4).

**Fig. 5:**
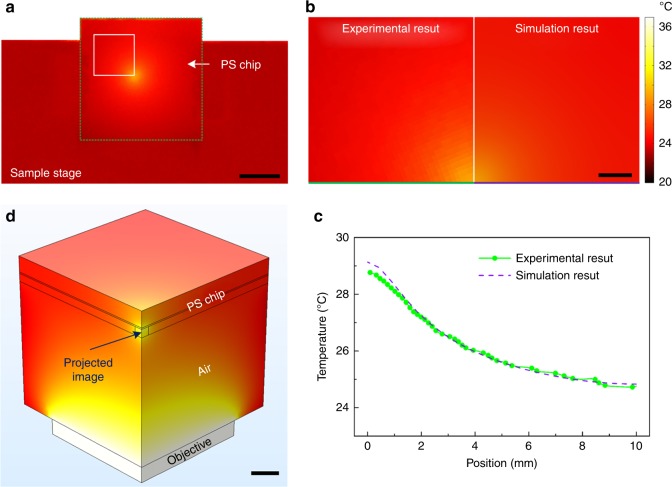
**a** Temperature distribution in the PS chip induced by illumination measured by an IR camera. The comparison between the experimental and simulated **b** temperature distribution and **c** temperature along the bottom line in **b**, **d**. Simulated temperature distribution around the PS chip corresponding to the marked area in **a**. Scale bars, 1 cm (**a**); 0.2 cm (**b**, **d**)

The internal three-dimensional temperature distribution of the OEK chip is difficult to directly measure using an IR camera. To map the temperature distribution in an OEK chip, we performed transient simulation by COMSOL. Because the temperature increase induced by illumination is more apparent than Joule heating, this simulation only considers illumination (Fig. 5). To match the experimental conditions, the temperature change in the objective over time measured by the IR camera (Supplementary Figure S4b) is imported into the simulation model. The simulated temperature distribution at 280 s (Fig. 5b, c) and the transient changes (Supplementary Figure S9) in the PS chip and the photoconductive substrate are highly consistent with the experimental results. Based on these simulations, we can acquire an accurate temperature distribution of the liquid inside the PS chip (Fig. 5d). When we add cell models to this simulation, the temperature distribution in cells in different areas of the PS chip can be determined. Supplementary Figure S10 shows that the maximum temperature difference in cells with a diameter of 20 μm can reach 0.7 °C for those around the illumination area and decreases from the lighted middle area to the surrounding region. This temperature gradient will induce thermophoresis in the cell, influencing molecular movement across the cytoplasm^37^. The temperature gradient along the PS chip will also influence the viability and activity of cells located in different positions^31^. Therefore, for biological applications, the light pattern must be properly designed to generate heat satisfying the cell requirements, and the temperature gradient must be decreased to minimize negative effects, e.g., thermophoresis. A heat sink or a stage temperature controller may be required, in which case a reflective OEK system where the projected light images for manipulation are focused by the optical microscope objective is facilitated to realize this function^33,38^.

### Optically induced thermocapillary flow

Although the temperature gradient can potentially induce damage to biological samples, it can also play a positive role in non-biological manipulation, assembly and fabrication. Temperature gradients can create surface tension gradients, and therefore, thermocapillary effects have been harnessed using prefabricated resistive heating elements to manipulate liquid droplets^39^. However, this method lacks flexibility and shows low-resolution addressability. An optically absorbing liquid is required for thermal gradients generated by the absorption of a laser in a liquid with greater flexibility to achieve the proposed thermocapillary-driven bubble or droplet movement; however, the choice of liquid is confined by physical and chemical properties^40^. When a photoconductive substrate is illuminated with light, it generates a local temperature increase (Fig. 1d), thereby providing a flexible approach to modulate a local temperature gradient field on the substrate and in the liquid media above it using arbitrary projected light images. The manipulation of gas bubbles in oil^41^ or water^29^ was demonstrated using light-induced thermocapillary effects. The use of a photosensitive substrate instead of absorbing liquids improves flexibility because it makes the optically induced thermocapillary effect independent of the optical properties of the manipulated liquids^41^.

**Fig. 6:**
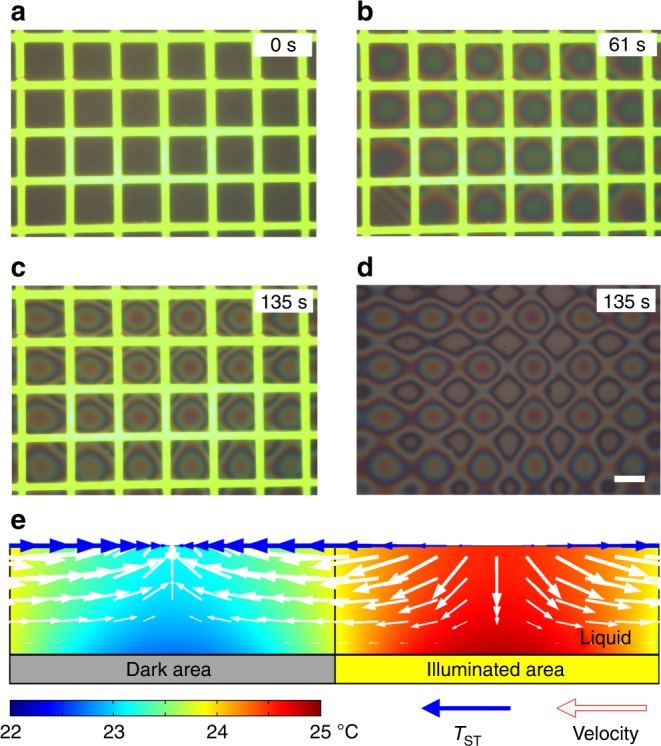
Optically induced thermocapillary flow for fluidic hydrogel patterning. **a**–**d** Experimental observation and **e** simulation study of optically induced thermocapillary flow. The color table represents the temperature distribution in the liquid layer. Scale bars, 50 μm (**d**)

Here, we propose that the patterning of thin hydrogel films can be realized by optically induced thermocapillary flow on a photoconductive substrate, which also demonstrates the temperature difference in PS or OEK chips. Initially, an ~1 μm poly(ethylene) glycol diacrylate (PEGDA, Mw = 10 kDa, Mn = 575; Sigma Aldrich) film is spun onto the photosensitive substrate. Optically induced thermocapillary flow can be triggered when 6.8 W/cm^2^ green light images illuminate the photosensitive substrate. A light grid image with period of ~75 μm and light width of ~14 μm is used in this experiment. For optically induced thermocapillary flow, external voltage is not applied. As shown in Fig. 6a–d, the hydrogel is driven from warmer (lighted area) to cooler (dark area) regions under the effect of interfacial shear stress (*T*_ST_) triggered by the light-induced temperature gradient *T*_ST_ = d*γ*/d*x* = (d*γ*/d*ϑ*) × (d*ϑ*/*dx*), where *γ* is the surface tension and ϑ is the temperature. As a result of this thermally induced liquid movement, the total surface energy is minimized. A multiphysics simulation of thermocapillary flow on photosensitive substrates is conducted using COMSOL. The simulated stationary solutions are shown in Fig. 6e. The shear stress at the interface between the hydrogel film and air and the velocity of the hydrogel fluidic layer point toward the dark areas from the illuminated areas consistent with the experimental observations. The patterned PEGDA can be further solidified by UV illumination, which has potential applications in the study of cell behavior^24^.

**Fig. 7:**
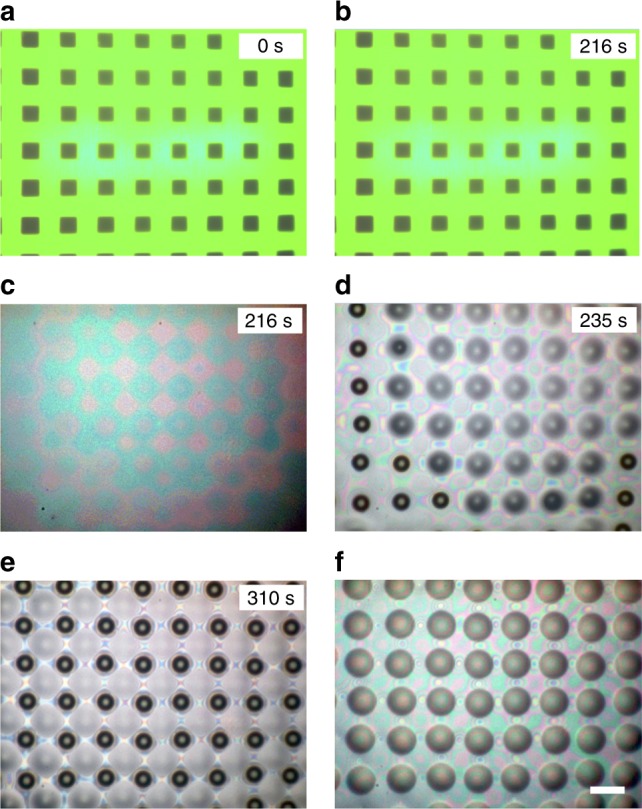
Microstructures fabricated by optically induced thermocapillary flow-assisted electrohydrodynamic instability. **a**, **b** A PDMS thick film (~1.7 μm) was illuminated by green light pattern. **c** After a 216-s illumination, a pre-pattern was observed when the projector was turned off. **d**, **e** Electrohydrodynamic instability was triggered at the nucleation sites generated by the thermocapillary flow. **f** Microlens array fabricated by separating the two electrodes in **d**, **e**. Scale bars, 80 μm (**f**)

Optically induced thermocapillary flow can also be used as pre-procedure to trigger other fluidic phenomena, e.g., electrohydrodynamic instability (EHDI) and to fabricate micro/nanostructures (Fig. 7). EHDI appears when an applied electric field overcomes surface tension in thin films that are spun onto a lower electrode and separated from an upper electrode by another medium (air, polymer or an ionic liquid)^10,11,42^. Initially, a polydimethylsiloxane (PDMS, Dow Corning, Sylgard-184) ~1.7-μm-thick film is spun onto the photosensitive substrate of the OEK chip, in which two aluminum foils are used to separate the two electrodes at a distance of ~11 μm. After a 216-s illumination by a projected green light pattern with intensity of 6.8 W/cm^2^ (Fig. 7a, b), a pre-pattern appears under the action of optically induced thermocapillary flow (Fig. 7c) in the PDMS layer, which generates instability nucleation sites in the dark areas. The illumination is turned off at 216 s, and electrohydrodynamic instability is triggered at the instability nucleation sites when a 250-V direct current (DC) voltage is applied by a voltage source (Keithley 2410). After 96 s, a micropillar array can be generated at the nucleation sites as the electrical forces (*N*_elec_) overcome surface tension (Fig. 7e). ($N_{{\mathrm{elec}}} = {\mathbf{n}} \cdot {\mathbf{m}} \cdot {\mathbf{n}} = \left\| {\varepsilon \varepsilon _0\left( {{\mathbf{E}} \cdot {\mathbf{n}}} \right)^2 - \varepsilon \varepsilon _0\left( {{\mathbf{E}} \cdot {\mathbf{t}}} \right)^2} \right\|/2$, where **n** and **t** are the local outer normal and tangent vectors on the interface, **E** is the electric field, *εε*_0_ represents the dielectric permittivity, and ‖‖ denotes the jump of operand across the interface.). Separating the two electrodes or applying a pulse instead of continuous DC voltage can facilitate the fabrication of a microlens array (Fig. 7f and Supplementary Figure S11). This process also demonstrates the optically induced thermocapillary function in the optically induced electrohydrodynamic instability proposed previously by our group^10,11,21^.

## Conclusions

Similar to DEP chips, in which the Joule heating can be minimized by optimizing the microelectrode geometry^35^, the projected light patterns must be properly designed to control the temperature distribution in an OEK chip, especially in a parallel manipulation system^5,6,8,26,43^ where complex light images cover a large chip surface area. The average temperature in the projected area is influenced by the light color, the total illumination area and the ratio of lighted regions to the total controlled areas. The situation is worsened when illumination and AC voltage are applied simultaneously because of the optically induced change in OEK chip electrical properties. The design of the light pattern needs to satisfy different application requirements. For biological applications, the temperature must be controlled to match physiological conditions, thereby requiring the use of an adequate heat sink or a temperature controller. However, cases involving, e.g., the polymerase chain reaction^44^, temperature gradient focusing^45^, and optically induced thermocapillary flow require high accuracy and resolution in temperature values or distribution^46^. Objectives with larger magnification or numerical aperture may be required to generate more intensively focused light images with higher spatial resolution. However, as the magnification or numerical aperture increases, the objective working distance decreases and, therefore, the objective thermal effect on OEK chip temperature will be more serious. We expect that the experimental results presented in this paper will expand the applications of OEK technology in different fields, especially in bio-related applications.

## Electronic supplementary material

Supplemental Information
