# Supplementary material for: Thermometry of photosensitive and optically induced electrokinetics chips
Source: Microsyst Nanoeng. 2018 Aug 27;4:26. doi: 10.1038/s41378-018-0029-y (PMC6220187; doi:10.1038/s41378-018-0029-y)
Supplement: Supplementary file 1 — Supplemental Information [file 41378_2018_29_MOESM1_ESM.docx]

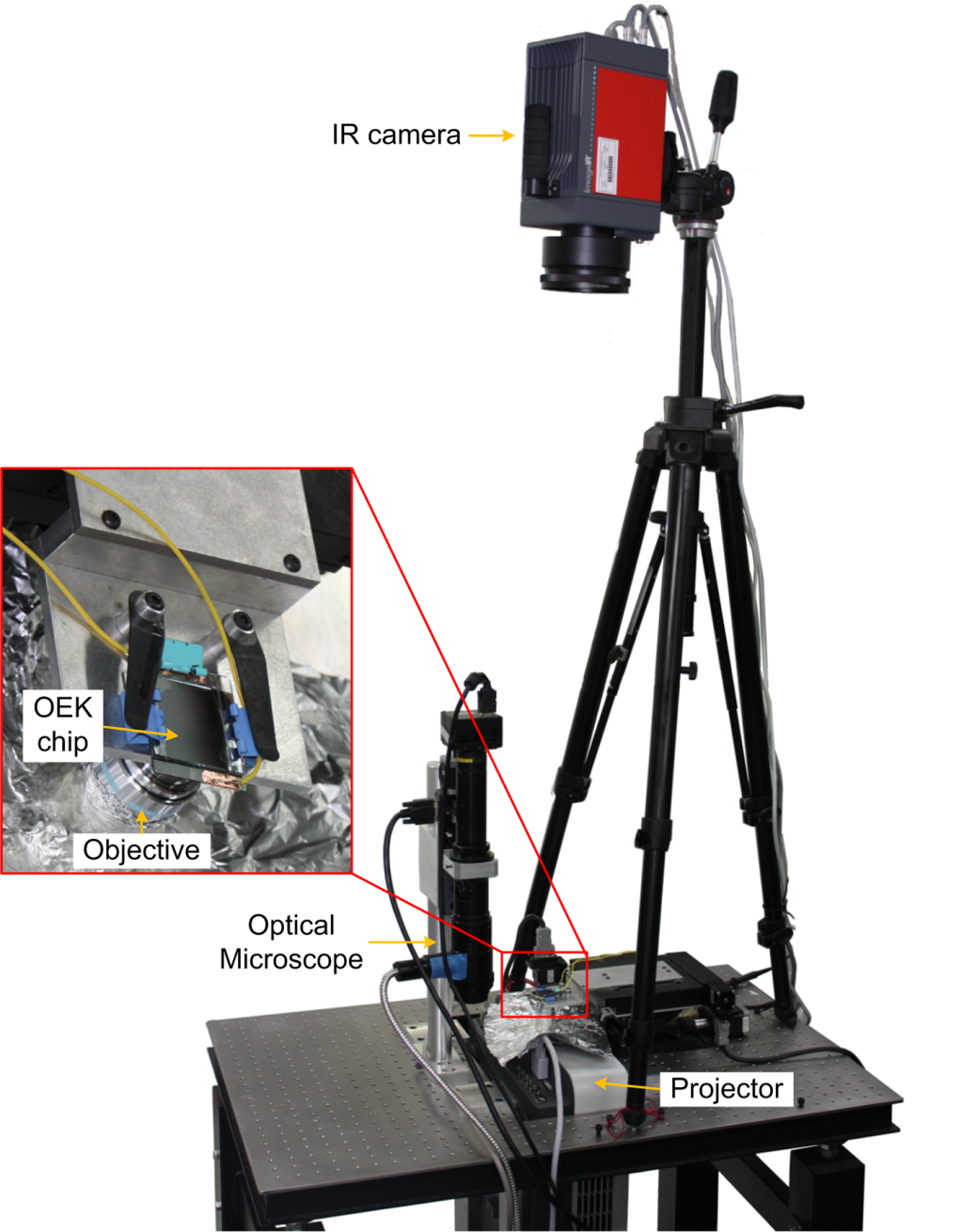


Supplementary Figure S1. Photograph of the experimental setup used to measure the chip temperature with an IR camera.





Supplementary Figure S2. Comparison between the temperatures of the temperature-controllable hotplate measured by IR camera and thermocouple.


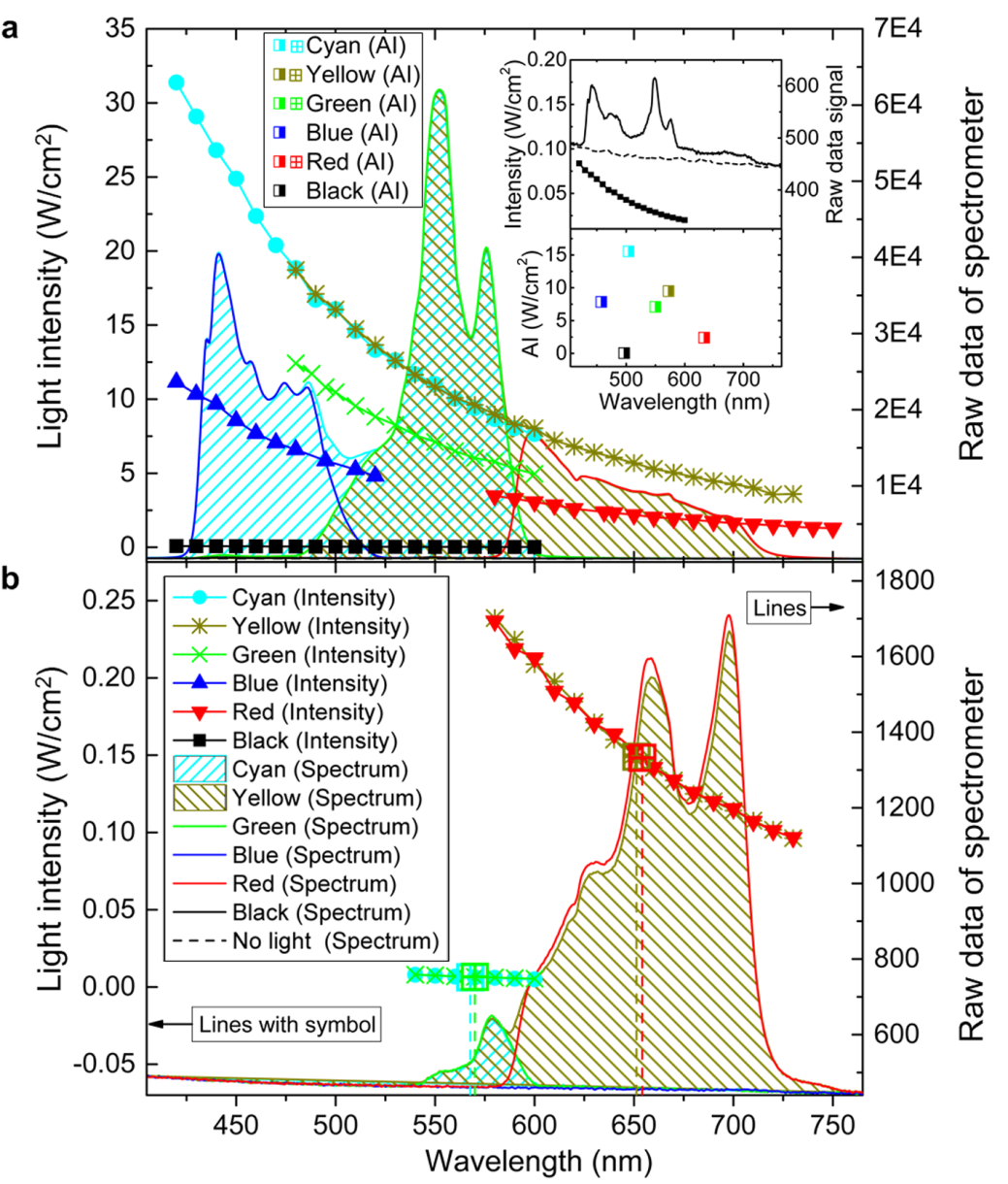


Supplementary Figure S3. Spectral measurement of the projector-generated illumination (a) before or (b) after transmission through a photosensitive chip. The inset shows the spectrum of the black color (upper) and the average light intensity of the projected light with different colors (lower).


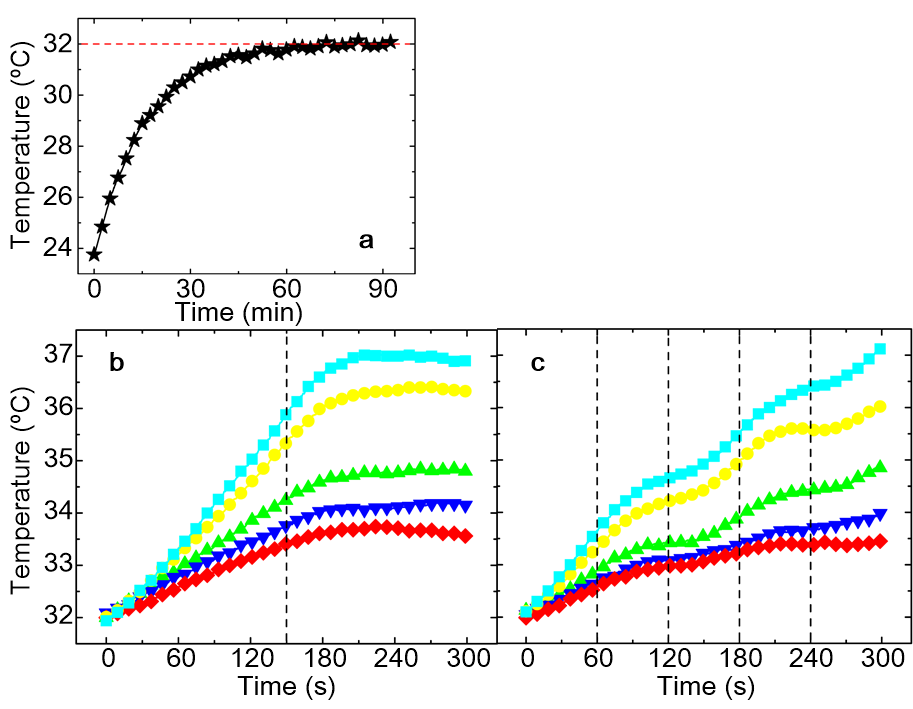


Supplementary Figure S4. The temperature changes in the objective caused by illumination. (a) Long-term temperature measurement of the objective illuminated by “black” light generated by the projector. Projected illumination is switched to “black” light for a duration of (b) 150 s or (c) 60 s.


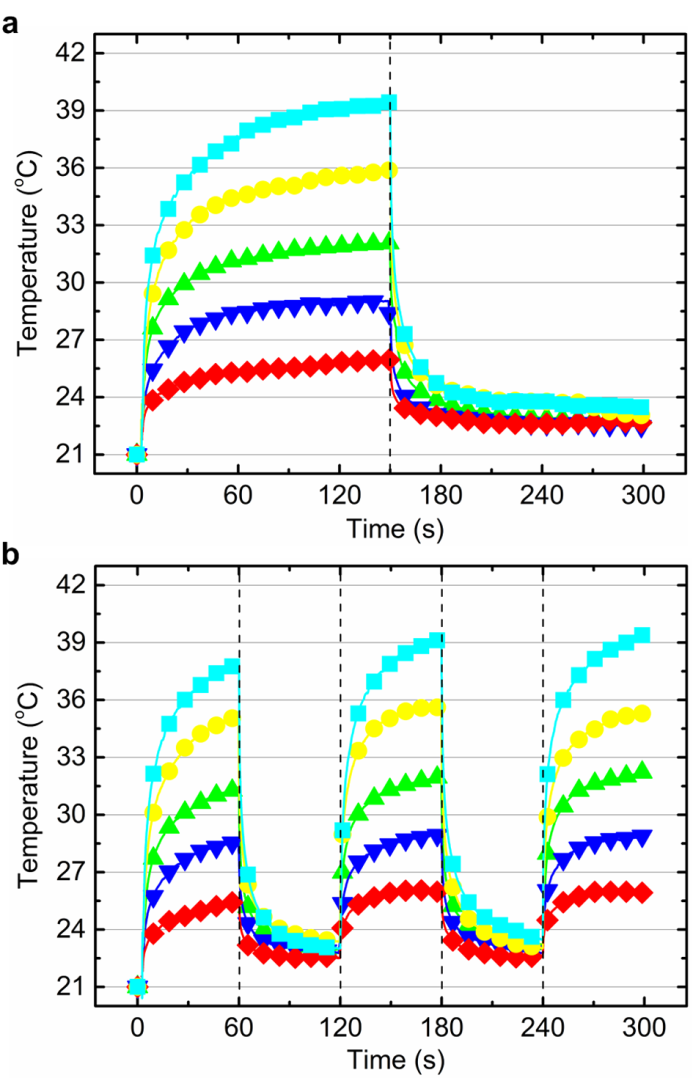


Supplementary Figure S5. Chip temperature changes as the illumination is switched to “black” light for a duration of 150 s or 60 s.


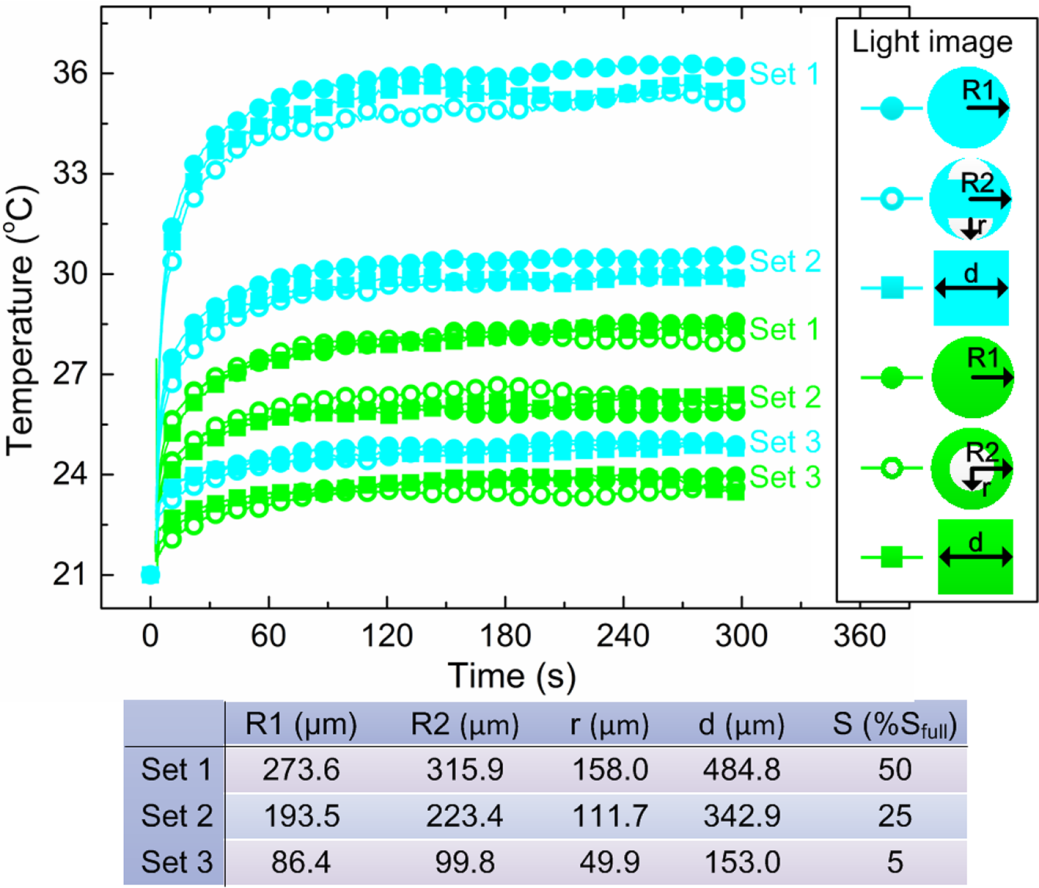


Supplementary Figure S6. Influence of the image shapes on the chip temperature.


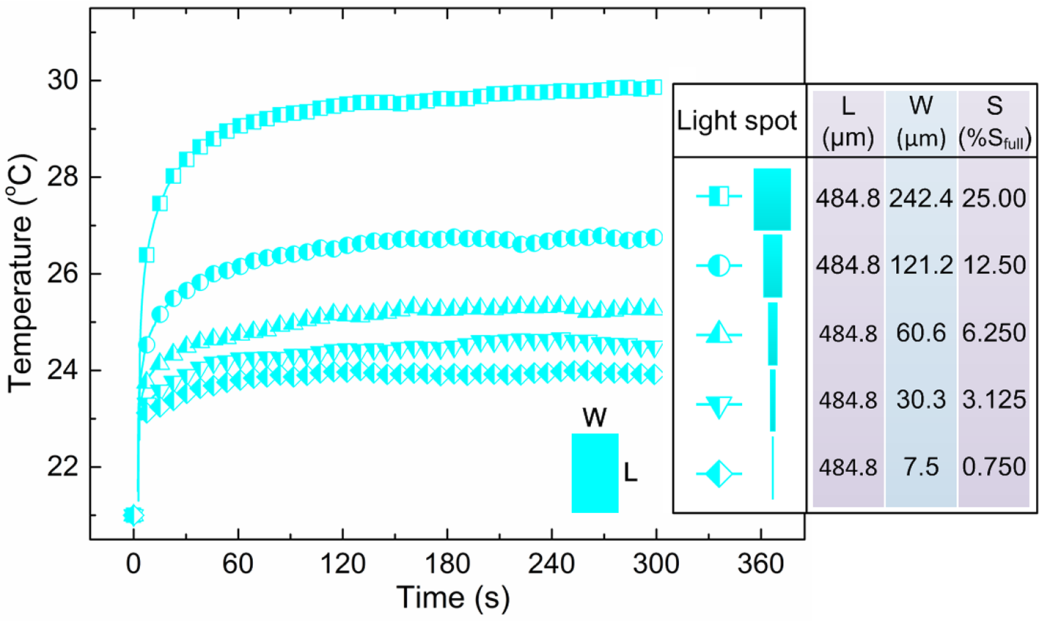


Supplementary Figure S7. Influence of the image size on the chip temperature.


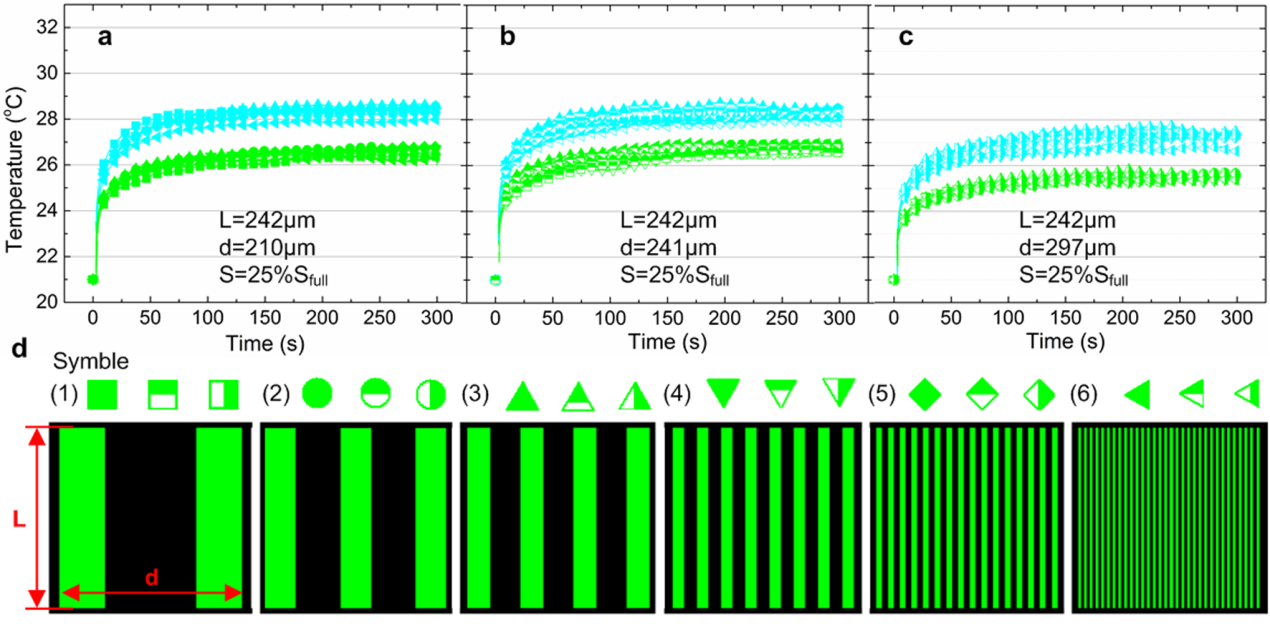


Supplementary Figure S8. Influence of the image distribution on the chip temperature.


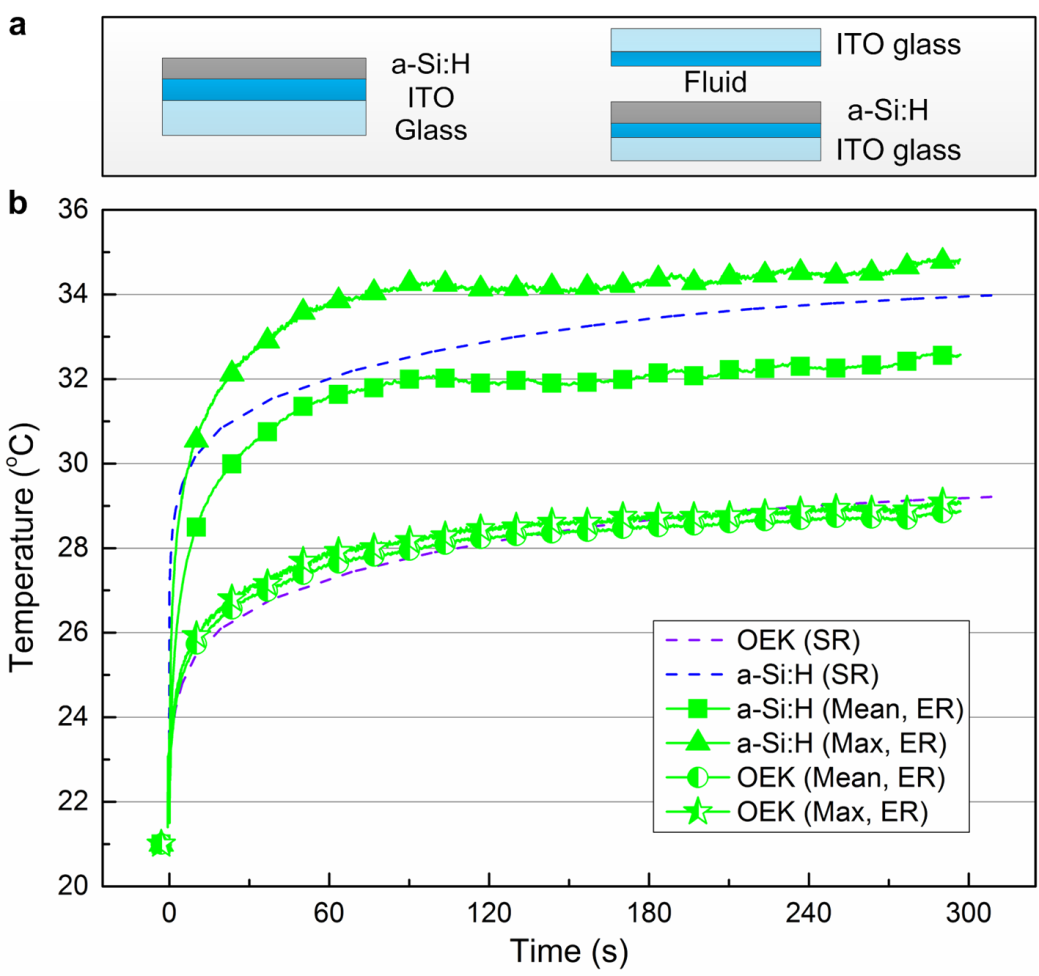


Supplementary Figure S9. Comparison between the experimental (ER) and simulation results (SR) of the temperature of the photosensitive substrate and the OEK chip.


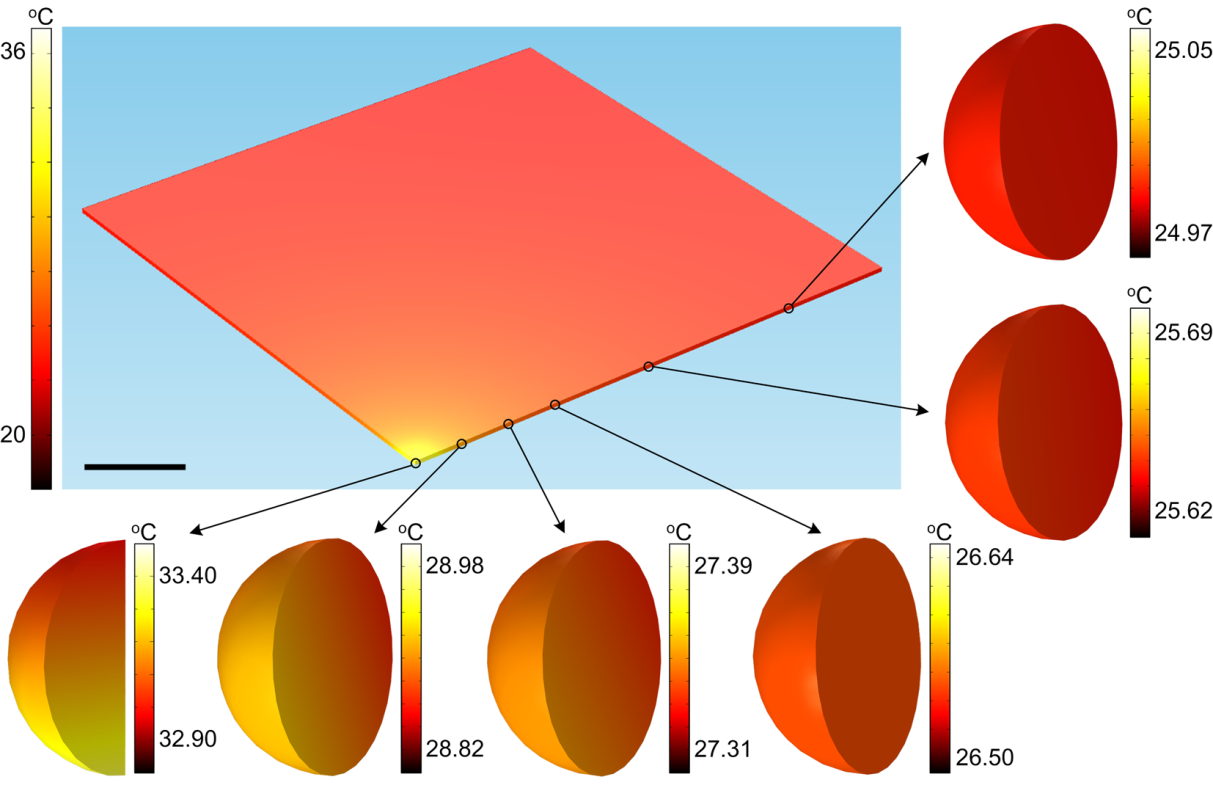


Supplementary Figure S10. The simulated temperature of cells distributed in different areas of the PS chip induced by illumination. The cell diameter in these simulations is 20 μm. Scale bars, 0.2 cm.


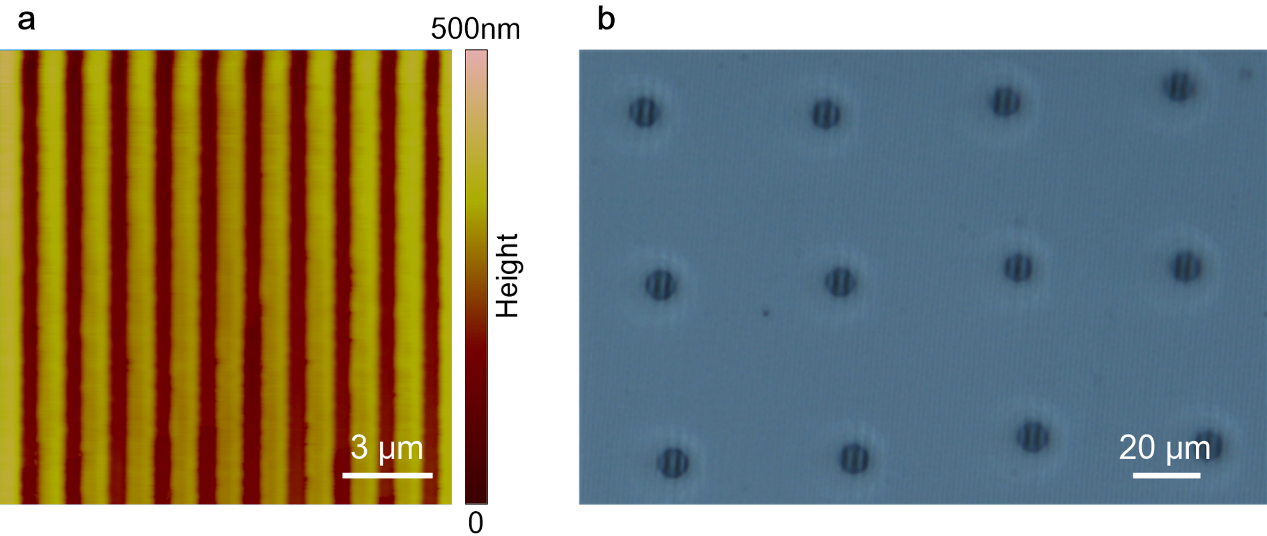


Supplementary Figure S11. Microstructure imaging by microlens array. (a) AFM scanning of a compact disc (CD) surface. (b) Image of a CD surface using a microlens array with diameter of ~ 10 µm. A Nikon microscope (Ti-U Nikon, Japan) fitted with a 50 x (numerical aperture = 0.6) objective and a CCD of DS-Fi1c (Nikon, Japan) was utilized.
